# RURAL OLDER ADULT CARE TRANSITIONS: A SCOPING REVIEW

**DOI:** 10.1093/geroni/igae098.4253

**Published:** 2024-12-31

**Authors:** Amiritha Kumar, Daniel Liebzeit

**Affiliations:** The University of Iowa, Iowa City, Iowa, United States; The University of Iowa, Iowa City, Iowa, United States

## Abstract

Older adult (age 65 and older) care transitions are frequent and complex, particularly in rural areas lacking equitable access to health and other services. This scoping review: 1) evaluates research on older adult rural care transitions and 2) synthesizes types of transitions studied, challenges and opportunities in rural health care, and gaps in the literature. Of a total of 6,976 articles retrieved in July 2024 from PubMed, CINAHL, PsycINFO, Dissertations and Theses, AgeLine, and Embas, 91 articles were included. Eligible articles focused on older adults, rural setting, a care transition, and provided empirical data. Findings revealed a primary focus on acute care transitions; care transitions involving long-term care and assisted living were vastly underrepresented. Most were observational, with few interventions to address the specific challenges faced by rural older adults. The evidence base on acute care transitions reveals disparities in readmission rates between rural and other populations, with limited focus on patient-centered outcomes. Limited literature on long-term care and assisted living transitions primarily focused on losses experienced, such as loss of independence. Although improved outcomes are often associated with collaborative, individualized planning, significant gaps remain in application of effective models of transitional care. Follow-up or post-acute care appears key for rural older adults, offering benefits comparable to urban settings; however, rural access barriers underscore the need for intervention development to reduce health disparities. Developing patient-centered interventions for rural older adults during care transitions is crucial, and addressing research gaps will create opportunities to further reduce disparities and improve outcomes.

